# Transcriptional Profiling of Canker-Resistant Transgenic Sweet Orange (*Citrus sinensis* Osbeck) Constitutively Overexpressing a Spermidine Synthase Gene

**DOI:** 10.1155/2013/918136

**Published:** 2012-12-27

**Authors:** Xing-Zheng Fu, Ji-Hong Liu

**Affiliations:** ^1^Key Laboratory of Horticultural Plant Biology (MOE), National Key Laboratory of Crop Genetic Improvement, College of Horticulture and Forestry Sciences, Huazhong Agricultural University, Wuhan 430070, China; ^2^Citrus Research Institute, Chinese Academy of Agricultural Sciences, Southwest University, Chongqing 400712, China

## Abstract

Citrus canker disease caused by *Xanthomonas citri* subsp. *citri* (Xcc) is one of the most devastating diseases affecting the citrus industry worldwide. In our previous study, the canker-resistant transgenic sweet orange (*Citrus sinensis* Osbeck) plants were produced *via* constitutively overexpressing a spermidine synthase. To unravel the molecular mechanisms underlying Xcc resistance of the transgenic plants, in the present study global transcriptional profiling was compared between untransformed line (WT) and the transgenic line (TG9) by hybridizing with Affymetrix Citrus GeneChip. In total, 666 differentially expressed genes (DEGs) were identified, 448 upregulated, and 218 downregulated. The DEGs were classified into 33 categories after Gene ontology (GO) annotation, in which 68 genes are in response to stimulus and involved in immune system process, 12 genes are related to cell wall, and 13 genes belong to transcription factors. These genes and those related to starch and sucrose metabolism, glutathione metabolism, biosynthesis of phenylpropanoids, and plant hormones were hypothesized to play major roles in the canker resistance of TG9. Semiquantitative RT-PCR analysis showed that the transcript levels of several candidate genes in TG9 were significantly higher than in WT both before and after Xcc inoculation, indicating their potential association with canker disease.

## 1. Introduction

Citrus canker disease caused by a biotrophic bacterium *Xanthomonas citri *subsp.* citri* (Xcc) is one of the most devastating diseases in many citrus-producing regions. This disease results in defoliation, dieback, and premature fruit drop, leading to enormous loss of yield and fruit quality [1, 2]. Once the canker-free citrus producing areas are invaded by Xcc, all the suspected and infected trees should be uprooted and burned *via* an eradication programme, as has been done in Florida [3]. Because of the serious destruction and recalcitrance to management, citrus canker has been regarded as a quarantine disease in many countries. Every year, millions of dollars are spent on prevention, quarantines, eradication programs, and disease control in the world [3]. 

Current strategies for counteracting with canker disease are primarily directed to integrated approaches such as eradication programme and use of antibiotics or bactericides [2]. However, due to the disadvantages in labor investment, safety, consistency, and stabilization these strategies are not the ultimate solutions. Moreover, their applications are often compromised by inducing adverse environmental influence and change of pathogen strains. Therefore, the most effective and economical approach for controlling canker disease relies on the production of resistant cultivars. Genetic manipulation *via* transforming stress-related genes is a widely employed way to create disease-resistant germplasms that are otherwise impossible for classic breeding programme, especially in citrus. At present, antibacterial peptides, *R*-genes, pathogenic factors, and defense-related genes have been applied to create canker-resistant germplasms in citrus [4, 5]. In our previous study, we produced transgenic sweet orange plants with less susceptibility to citrus canker *via* constitutively overexpressing a spermidine synthase (SPDS, EC 2.5.1.16) [6].

SPDS is a key enzyme involving in polyamine biosynthetic pathway, which converts putrescine (Put) to spermidine (Spd). Polyamines are low-molecular-weight aliphatic compounds that exist ubiquitously in all living organisms, mainly including diamine Put, triamine Spd, and tetraamine spermine (Spm). It has been well documented that polyamines are closely involved in a variety of physiological processes, including biotic stress responses. For example, the content of free and conjugated polyamines and the activities of polyamine biosynthetic and oxidative enzymes increased during the hypersensitive response (HR) of barley after the powdery mildew fungus attack [7], as well as during the formation of maize tumors induced by the the biotrophic pathogenic fungus *Ustilago maydis* [8]. The transcript levels of polyamine biosynthesis-related genes were also found to be accumulated in TMV-infected tobacco [9] and in *U. maydis*-infected maize [8]. In tobacco, polyamine oxidase (PAO) protein and the specific PAO enzymatic activities increased after infection with compatible plant-pathogenic bacterium *Pseudomonas syringae* pv *tabaci* [10]. The studies indicated that polyamines or PAO protein accumulation may be a common event for plant response to pathogens. Moreover, augmentation of endogenous polyamine level by exogenous application of polyamine enhanced host resistance to virus or to bacterial challenge [11–13]. The previous work provided numerous evidence showing that polyamines play important roles in plant pathogen responses. 

The mechanisms underlying the role of polyamines in plant defense have been described in previous studies. In summary, two main mechanisms have been proposed. The first one relates to production of hydrogen peroxide (H_2_O_2_) due to PAO-mediated polyamine catabolism, triggering HR and induced tolerance to specific pathogens [6, 8–10, 12]. The second one points to the role of polyamines (especially for Spm) as signaling molecules to activate expression of pathogenesis-related proteins [14] and a subset of HR-specific genes [15, 16]. Mitsuya et al. [14] and Sagor et al. [17] found that a number of genes in *Arabidopsis* showed response to exogenous application of Spm and *Cucumber mosaic virus* (CMV) infection based on the super serial analysis of gene expression (SuperSAGE), implying that Spm-mediated signaling pathway might play a role in CMV response. Very recently, Gonzalez et al. [18] reported that a large number of differentially expressed genes were identified in Spm-overproducing transgenic *Arabidopsis* and Spm-decreased mutant by using microarray analysis. It should be pointed out that although many efforts have been invested, the underlying physiological and molecular mechanisms remain still elusive. The gene regulation network in polyamine involved plant pathogen response is largely unclear, particularly in perennial plants like citrus. In our previous study, ectopic expression of *SPDS* gene increases both Spd and Spm levels in the transgenic sweet orange and confers canker resistance. As Spm functions as a signaling molecule, it is hypothesized that the transgenic line might display an extensive transcriptional reprogramming. To address this issue and to gain new insights into the molecular mechanisms on the enhanced disease tolerance, genome-wide transcriptome analysis was conducted using the Affymetrix Citrus GeneChip microarray technology. The Affymetrix Citrus GeneChip contains 30,171 probe sets representing up to 33,879 citrus transcripts selected from citrus HarvEST EST and cDNA clustering database. The transcriptional profiling described here may contribute to explain molecular mechanism of polyamine in regulating plant pathogen response.

## 2. Methods and Materials

### 2.1. Microarray Hybridization and Data Analysis

The leaves were collected from untransformed line (WT) and the transgenic line (TG9) plant for hybridization with the Affymetrix Citrus GeneChip (Affymetrix, Santa Clara, CA, USA). In brief, 2 g leaves were sampled from uniform new flushes (about 20 days after sprout) of WT and TG9, and then immediately immersed in liquid nitrogen and stored at −80°C. All the other processes including the total RNA extraction (20 *μ*g at least), cDNA and cRNA synthesis, cRNA fragmentation, hybridization, washing and staining, and scanning were performed by Gene Technology Company Limited of Shanghai in China. The detailed experimental procedures can be found in the GeneChip Expression Analysis Technical Manual (http://www.affymetrix.com/support/downloads/manuals/expression_analysis_technical_manual.pdf). To satisfy biological reproducibility requirements, the experiment was carried out using three independent biological replicates for both WT and TG9 (means both WT and TG9 were hybridized with microarray for three times).

The probe array was scanned with the Affymetrix GeneChip Scanner 3000, and the images were analyzed with the Affymetrix GeneChip Operating software (GCOS 1.4) to generate raw data, saved as CEL files. The CEL files were then imported into commercial Partek Genomic Suite 6.4 software (Partek Inc., St. Louis, MO) according to the way of RMA quantile normalization to obtain RMA data containing the expression values. Analysis of variance (ANOVA) was used to compare the statistical expression difference between TG9 and WT. Probe sets with a *P* value ≤ 0.05 and 2-fold change were considered as differentially expressed genes (DEGs) between the two groups at a statistically significant level.

### 2.2. Microarray Annotation and Functional Analysis

To assign putative functions of DEGs, Gene ontology (GO) term, Enzyme Commission (EC), and Kyoto Encyclopedia of Genes and Genomes (KEGG) annotation were performed using the Blast2GO [19] software. Blast2GO assigns GO annotation through three steps, blasting, mapping, and annotation. GO terms for each of the three main categories (biological process, molecular function, and cellular component) were obtained by using the combined graphs function of the software with default parameters. The KEGG analysis were performed by using the KEGG annotating function of Blast2GO software, and the annotated KEGG pathways were further manually classified according to the published KEGG pathway lists (http://www.genome.jp/kegg/pathway.html).

### 2.3. Semiquantitative RT-PCR Analysis

Semiquantitative RT-PCR was employed to validate the microarray results using the same set of RNA samples for the hybridization experiments. Each RNA sample was pretreated with PCR amplification-grade RNase-free DNase I (Takara, Dalian, China) at 37°C to exclude DNA contamination. cDNA synthesis was done by the ReverTra Ace-*α*-kit (Toyobo, Japan) following the manufacturer's instructions. Specific primers of candidate genes were designed by Primer Premier 5.0 software (PRIMER Biosoft International, Palo Alto, CA) based on the citrus consensus sequences downloaded from Affymetrix website (Table 1). Each PCR reaction was composed of 200 ng cDNA, 2.0 *μ*L 10× reaction buffer, 1.0 mM MgCl_2_, 0.2 mM dNTP, 1.0 U of DNA polymerase (*Taq*, Fermentas) and 0.4 *μ*M of each primer in a total volume of 20 *μ*L. PCR amplifications were performed at 94°C for 5 min, followed by 28–32 cycles of 94°C for 40 s, 52°C for 40 s, 72°C for 40 s and 5 min extension at 72°C. An *Actin* gene (Table 1, [20]) was used as an internal positive control. Band intensity was quantified by Quantity One analysis software (Bio-Rad Laboratories), and the fold change was calculated by the signal intensity of TG9-specific product divided by the signal intensity of WT-specific product.

In another experiment, semiquantitative RT-PCR was performed to evaluate the expression patterns of several genes before or after *Xanthomonas axonopodis* pv. *citri* (Xcc) inoculation. For this purpose, the leaves sampled from uniform new flushes (about 20 days after sprout) of WT and TG9 were divided into two groups, respectively. One group of leaves without Xcc inoculation (uninoculated leaves) were immediately immersed in liquid nitrogen and stored at −80°C. And another group of leaves were subjected to a pinprick inoculation with Xcc bacterial suspension as described by Fu et al. [6]. Twenty-four hours after inoculation (hpi), the whole leaves of WT and TG9 were collected and stored at −80°C. The total RNA was isolated from uninoculated (0 hpi) and inoculated (24 hpi) leaves according to Liu et al. [21]. The other processes including RNA pretreatment, cDNA synthesis, PCR amplification, and quantification of band intensity were the same as mentioned above.

## 3. Results

### 3.1. Screening of the Differentially Expressed Genes and Verifying the Microarray Data

In our previous study, we produced a *SPDS*-overexpressed transgenic sweet orange line with higher levels of Spd and Spm and better resistance to canker disease [6]. To reveal the molecular mechanisms underlying canker resistance in TG9, the global transcriptional profiling of TG9 and WT were compared by citrus genome Genechip analysis. After statistical analysis, 666 genes with signal ratio fold change larger than 2 or smaller than 0.5 (*P* value ≤ 0.05) between the TG9 and WT were identified as differentially expressed genes (DEGs). Among these genes, 448 and 218 were upregulated and downregulated, respectively (see Supplemental Tables S1 and S2 in Supplementary Material available online at doi:10.1155/2012/918136).

**Figure 1:**
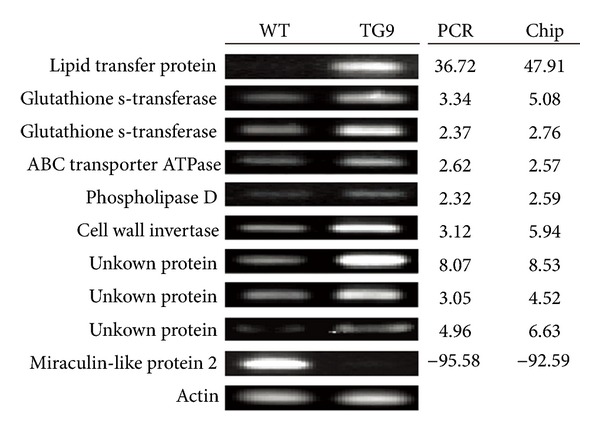
Validation of microarray results by semiquantitative RT-PCR. Ten probes putatively encoding lipid transfer protein (Cit.60.1.S1_at), glutathione s-transferase (Cit.6308.1.S1_at, Cit.9510.1.S1_s_at), ABC transporter ATPase (Cit.3246.1.S1_at), phospholipase d (Cit.6097.1.S1_s_at), cell wall invertase (Cit.5734.1.S1_at), miraculin-like protein 2 (Cit.57.1.S1_at) and unknown protein (Cit.19313.1.S1_at, Cit.5367.1.S1_at, Cit.1406.1.S1_s_at) were amplified with specific primers in WT and TG9 leaves, using *Actin* gene as an internal control for examining equal cDNA loading. The expression ratios between TG9 and WT were calculated by quantifying the band density using the Quantity One software.

In order to verify the reliability of the microarray data, 9 upregulated and 1 downregulated genes were randomly selected to analyze their expression levels in TG9 and WT *via* semiquantitative RT-PCR using gene-specific primers (Table 1). These genes putatively encode lipid transfer protein, glutathione s-transferase, ABC transporter ATPase, phospholipase d, cell wall invertase, miraculin-like protein 2, or unknown protein. As shown in Figure 1, transcript levels of the upregulated and the downregulated genes in TG9 were significantly higher or lower than in WT. The fold changes of these genes based on the calculation from semiquantitative RT-PCR results were largely consistent with the microarray data, suggesting that the microarray data are reliable.

### 3.2. Functional Annotation and Classification of the Differentially Expressed Genes

To further analyze the microarray data, the identified DEGs, including significantly upregulated and downregulated genes, were functionally annotated and classified using Blast2GO software. The annotated information of each gene such as sequence description, accession number of blasted gene, GO term annotation was listed in Supplemental Tables S1 and S2. The genes putatively encoding cysteine proteinase inhibitor (Cit.8163.1.S1_x_at, Cit.30421.1.S1_s_at, Cit.28011.1.S1_x_at) had the highest fold change (as high as 268.12), and the others such as lipid transfer protein (Cit.60.1.S1_at), lipid binding protein (Cit.19161.1.S1_at) and cytochrome p450 (Cit.26116.1.S1_at, Cit.4425.1.S1_at) were also upregulated to a high level (Table S1). For the downregulated genes (Table S2), the gene with the maximum fold change was miraculin-like protein. The other downregulated genes with high fold change include DNA binding protein (Cit.8142.1.S1_at, Cit.30420.1.S1_x_at), early light-inducible protein (Cit.165.1.S1_s_at), and AP2/ERF domain-containing transcription factor (Cit.11068.1.S1_at, Cit.30607.1.S1_s_at). 

**Figure 2:**
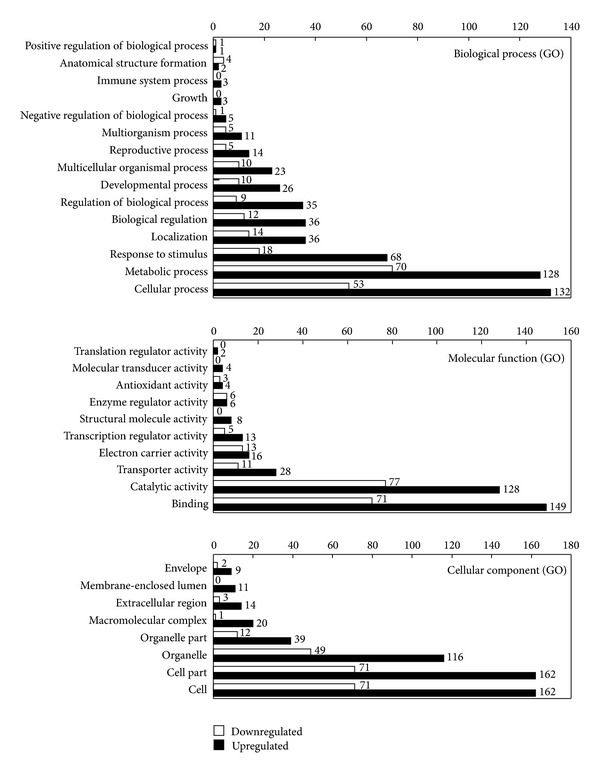
Functional categorization of upregulated and downregulated differentially expressed genes. 448 significantly upregulated and 218 significantly downregulated DEGs were categorized to biological process, molecular function, and cellular component based on GO annotation, and the represented number of each column was marked in the figure.

The functional categorization was performed according to biological process, molecular function, and cellular component using Blast2GO software. As shown in Figure 2, the biological processes of these DEGs included mainly 15 categories such as cellular process, metabolic process, response to stimulus, localization and biological regulation, and among which the genes in response to stimulus and immune system process are of interest because they may participate in canker disease resistance directly. In addition, it is intriguing to find that most of these categories contained larger number of the upregulated genes than the downregulated genes, such as cellular process, metabolic process, response to stimulus, and so forth. In the immune system process, only upregulated genes were assembled to this group. Molecular functions were primarily related to binding activity, catalytic activity, transporter activity, electron carrier activity, transcription regulator activity, and others (Figure 2). Cellular component included cell, organelle, macromolecular complex, extracellular region, membrane-enclosed lumen, and envelope. Similar to the biological process category, in molecular function and cellular component the number of upregulated genes is larger than that of downregulated genes (Figure 2).

### 3.3. The Expression of Candidate Genes before and after Inoculation

According to the functional annotation and classification of the DEGs, and related studies in previous literatures, 11 genes putatively encoding cysteine proteinase inhibitor, thaumatin-like protein, cytochrome p450, aspartyl protease family protein, pyruvate kinase, pathogenesis-related protein, thioredoxin-like 5, AP2/ERF transcription factor, NADPH oxidase, TIR-NBS-LRR resistance protein, and Rubisco subunit binding protein were hypothesized to be involved in canker resistance of TG9. To answer this question and to identify canker responsive genes, the expression levels of these candidate genes were evaluated before (0 hpi) and 24 h after Xcc inoculation (24 hpi).

**Figure 3:**
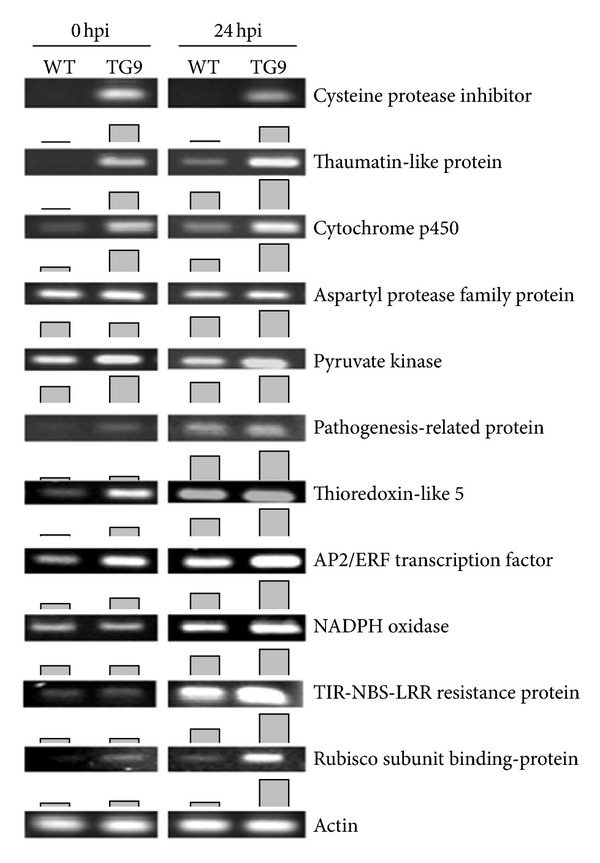
Expression of candidate genes in leaves of WT and TG9 infected or not with *Xanthomonas citri* subsp. *Citri* (Xcc). Eleven candidate genes putatively encoding cysteine proteinase inhibitor (Cit.8163.1.S1_x_at), thaumatin-like protein (Cit.11548.1.S1_at), cytochrome p450 (Cit.4425.1.S1_at), aspartyl protease family protein (Cit.28117.1.S1_s_at), pyruvate kinase (Cit.13055.1.S1_at), pathogenesis-related protein (Cit.20495.1.S1_at), thioredoxin-like 5 (Cit.5856.1.S1_at), AP2/ERF transcription factor (Cit.17124.1.S1_at), NADPH oxidase (Cit.2333.1.S1_at), TIS-NBS-LRR resistance protein (Cit.6121.1.S1_at), and Rubisco subunit binding protein (Cit.31932.1.S1_at) were assessed at 0 and 24 h post inoculation (hpi) in WT and TG9 leaves *via* semiquantitative RT-PCR. Column diagram was the quantification data of corresponding bands using Quantity One software.

Before inoculation higher expression levels of the tested genes were detected in TG9 than in WT except those encode aspartyl protease family protein, NADPH oxidase, and TIS-NBS-LRR resistance protein which showed no difference between TG9 and WT (Figure 3). However, at 24 hpi the expression levels of all genes were higher in TG9 than in WT. Moreover, it is interesting to see that Xcc inoculation upregulated the genes in both TG9 and WT as compared with absence of Xcc inoculation, such as thaumatin-like protein, cytochrome p450, pathogenesis-related protein, thioredoxin-like 5, AP2/ERF transcription factor, NADPH oxidase, TIS-NBS-LRR resistance protein, and Rubisco subunit binding protein (Figure 3). Of note, the gene encoding cysteine proteinase inhibitor expressed at high levels in TG9 with or without Xcc inoculation, but it was not detected in the WT (Figure 3). Our data suggested that the expression of these candidate genes were constitutively upregulated in the transgenic line and can be further induced by the Xcc inoculation, which provides important information and evidence for its potential role in canker disease resistance.

## 4. Discussion

In our previous study, ectopic expression of a polyamine biosynthetic gene (*MdSPDS1*) in sweet orange confers citrus canker resistance, and transcript levels of several defense-related genes were induced in the transgenic line [6]. Therefore, we speculate that global transcriptional levels of transgenic plants are regulated due to the overexpression of *MdSPDS1*, which may explain at the transcriptional level the enhanced resistance in the transgenic plants. To confirm this hypothesis, global transcriptional profiling of WT and TG9 was compared through hybridizing with Affymetrix Citrus GeneChip in the present study. Genechip, a high-throughput and effective technology for studying global transcriptional profiling, has been widely used for deciphering molecular responses to abiotic and biotic stresses and comparing transcriptome under different treatments [22–24]. 

 In the current study, 666 genes were identified as DEGs, accounting for 1.97% of all transcripts in the citrus genechip. Among these DEGs, the number of upregulated genes was about twice as that of the downregulated ones, in line with the microarray data overexpressing a spermidine synthase gene in *Arabidopsis* [25]. This result indicated that overexpression of a polyamine biosynthetic gene may lead to more prominent induction of the global transcript level. To further understand these DEGs, the functional annotation and classification were conducted using Blast2GO software. Out of the DEGs 60.66% were annotated, 39.34% upregulated, and 21.32% downregulated. Those genes without an annotation, including NoBLAST, NoMapping, and NoAnnotation, may be attributed to scarcity of enough amount of information of selected database or parameter setting. The phenomenon is not distinct as it has been also reported in previous studies [26, 27]. After GO annotation, all these DEGs were classified into 33 categories involved in biological process, molecular function, and cellular component. Moreover, the KEGG pathways of upregulated DEGs were annotated for further understanding participant metabolic and cellular processes. Three categories and annotated KEGG pathways were subjected to more detailed discussion as follow. 

### 4.1. Genes Involved in Stimulus Response and Immune System Process

According to the GO annotation, 68 upregulated genes were shown to be involved in stimulus response and immune system process (Table 2). Based on this functional classification, we speculate that these genes have important relevance to canker disease. For instance, the gene (Cit.8878.1.S1_at) encoding major allergen pru had the highest fold change (8.63). This gene was also annotated as stress-related protein and pathogenesis-related (PR) protein 10, indicating that it may be associated with the pathogen defense. In this category, other genes include thaumatin-like protein, *β*-1,3-glucanase, AP2/ERF domain-containing transcription factor, ATP-binding cassette (ABC) transporter, copper/zinc superoxide dismutase, disease resistance protein, glutathione-*S*-transferase, and aspartyl protease family protein. 

Thaumatin-like protein (TLPs), categorized under the PR5 family, can be induced by various stresses, such as salinity, wound, and pathogen infection [28]. In addition, *in vitro* bioassays have shown that TLPs possess antifungal activity [29]. In the present study, expression analysis of TLPs before or after Xcc inoculation showed that TG9 had significant higher transcript levels than WT, in particularly after Xcc inoculation (Figure 3), indicating the function of TLPs on canker disease resistance. *β*-1,3-glucanase, hydrolyzing the 1,3-*β*-D-glucosidic linkages of *β*-1,3-glucan, belongs to PR2 family, has been shown to play a crucial role in plant pathogen defense [30–32]. AP2/ERF domain-containing transcription factor is an important plant-specific transcription factor, which has been suggested to play a critical role in stress response. Overexpression of AP2/ERF induced several PR genes expression and enhanced disease resistance in tobacco [33, 34]. In this study, AP2/ERF was induced by Xcc inoculation, and TG9 had a higher transcript level than WT (Figure 3), suggesting that AP2/ERF domain-containing transcription factor may be presumably implicated in citrus canker disease. ABC transporter, a membrane protein, exists in bacteria, fungi, animals, and plants and acts on absorption and secretion of many substrates. Martinoia et al. [35] reported that ABC transporter possibly involved in secretion of antimicrobial compounds and detoxification of some toxic metabolites in plant defence.

### 4.2. Cell Wall-Related Genes

Plant cell wall is a battlefield of host-pathogen interaction because the invasion of pathogen must first break the physical barrier of plant cell wall, which constitutes the first line of pathogen defence [36–38]. In this study, 12 cell wall-related genes were significantly upregulated in TG9, including cysteine proteinase inhibitor, basic 7s globulin 2 precursor small subunit, glutathione-*S*-transferase, and some hypothetical cell wall proteins (Table 3). Cysteine proteinase inhibitor, a proteinase inhibitor, is categorized under the PR6 family, which plays important roles in plant defence [28, 39]. As shown in Figure 3, the gene encoding cysteine proteinase inhibitor (Cit.8163.1.S1_x) was upregulated in TG9 before and after Xcc inoculation, but it was not detected in WT. Solomon et al. [40] reported that cysteine proteinase inhibitor can regulate the process of programmed cell death (PCD), an important step in HR. Therefore, cysteine proteinase inhibitor can possibly enhance disease resistance *via* regulating HR in plants, in agreement with an apparent HR of TG9 leaves in our previous work [6]. Among the 12 cell wall-related genes, Cit.14918.1.S1_at was annotated as basic 7s globulin 2 precursor small subunit (the similarity was 100%) by blast2go or annotated as xyloglucan-specific endoglucanase inhibitor protein (XEGIP, the similarity was 99%) in NCBI database. XEGIP has been shown to be the newest class of plant-derived proteins that inhibit pathogen-secreted cell wall degrading enzymes [41, 42]. Cernadas et al. [22] also reported that canker disease inoculation induced upregulation of endoglucanase inhibitor protein in sweet orange. Therefore, we can speculate that the gene XEGIP is possibly involved in canker disease resistance *via* suppressing the plant cell wall degradation in the transgenic line. Glutathione-*S*-transferase (GST), a protein with multiple functions, is closely associated with detoxification of some hydrophobic and electrophilic compounds, transport of auxin and phenylpropanoids, and activation of phenylpropanoid metabolism as signalling molecules [43, 44]. GST has been proposed as a marker gene for pathogen reactions, and an increase in transcript level of GST gene has been shown to be relevant to pathogen challenge [28]. In this study, the transgenic line had higher expression levels of GST, suggesting that the former may exhibit a better detoxification and regulation capacity under biotic stress when compared with the wild type, leading to less serious damage.

### 4.3. Transcription Factors Pertinent to Pathogen Attack

It is well known that transcription factors (TFs) play a crucial role in abiotic and biotic stresses *via* regulating a series of downstream target genes. In this study, 13 TFs were identified in the upregulated genes, including AP2/ERF, MADS, BT4 (BTB and TAZ domain protein 4) protein, NAC, MYB, and several other unnamed TFs (Table 4). In an earlier work, Kasukabe et al. [25] reported that TFs like AP2/ERF, NAC, and MYB were upregulated in the transgenic *Arabidopsis thaliana *plants overexpressing *FSPDS* gene relative to the wild type. Moreover, 47 TFs, such as NAC, MYB, WRKY, and bZIP, were found to be upregulated in SPMS-overexpressing transgenic *Arabidopsis* [18]. Our data and earlier results demonstrate that modification of the polyamine synthesis may cause the transcription reprogramming in the transgenic plants, which may be ascribed to the regulatory role of polyamines. TG9 contained higher level of spermine, which has been proposed as a signal molecule in previous studies [11, 14]. The upregulation of an array of the TFs suggests that the transgenic plants possess a robust system of transcriptional modulation towards the disease tolerance by regulating a large spectrum of relevant target genes of different TFs. Implication of the corresponding TFs in biotic stress has been experimentally corroborated in earlier studies [45–50]. For example, Nakashima et al. [48] reported transgenic rice transformed with an NAC gene displayed enhanced resistance to blight disease. In another work, Vailleau et al. [50] showed that overexpression of an *MYB* gene in *Arabidopsis thaliana* and tobacco conferred resistance to both bacteria and fungus. 

Apart from the abovementioned TFs, it is interesting to find that the TFs involved in flowering regulation, such as MADS and FLC (flowering locus C) [51], were also upregulated in the TG9. Upregulation of MADS and FLC in TG9 suggests that overexpression of *MdSPDS1* has led to alteration of gene network associated with flowering in the transgenic plant. Although it will need time to compare the flowering dynamics between the transgenic line and WT, the polyamines have been shown to participate in the physiological process of flowering in plants [52, 53]. 

### 4.4. Potential KEGG Pathways Involved in Defence

To further understand the metabolic and cellular processes involved in defence, KEGG pathways of the upregulated DEGs were annotated by Blast2GO software. As shown in Supplemental Table S3, the annotated KEGG pathways included carbohydrate metabolism, energy metabolism, lipid metabolism, nucleotide metabolism, amino acid metabolism, cofactors and vitamins metabolism, biosynthesis of polyketides, terpenoids, alkaloids, hormones and other secondary metabolites, and phosphatidylinositol signaling transduction process. 

Starch and sucrose not only serve as typical carbon and energy sources, but also play important roles in plant defense. Sucrose has been recognized as an endogenous signal to induce defense responses against pathogens [54]. Recently, Singh and Shah [55] reported that starch and sucrose contents significantly accumulated in the green peach aphid infested *Arabidopsis* and tomato leaves. Starch accumulation has been suggested to facilitate the host plant to generate a secondary sink that suppresses the insect to manipulate host metabolism [55]. Improved metabolism of starch and sucrose in TG9 possibly provides energy and signaling to the plant against Xcc attack. Over the past three decades, glutathione was gradually known to be involved in plant defense reactions and as a signaling molecule to induce various defense genes, and it has also been reported to crosstalk with a variety of hormone-related defense signaling, such as salicylic acid (SA), jasmonic acid (JA), ethylene (ET), and abscisic acid (ABA) [56]. Höller et al. [57] reported that enhanced glutathione metabolism was correlated with sulfur-induced resistance in *tobacco mosaic virus*-infected tobacco plants. Therefore, we could speculate that enhanced glutathione metabolism in TG9 is a potential mechanism for the enhanced Xcc resistance. On the other hand, biosynthesis of other compounds, such as cytochrome P450, phenylpropanoids, and plant hormones has been well documented to be involved in pathogen defense. For example, cytochrome P450 plays critical roles in the biosynthesis of defense-related compounds, hormones, and signaling molecules [58]. And it is interesting to find that a number of cytochrome P450-related genes were induced in Xcc-inoculated Meiwa kumquat (*Fortunella crassifolia*) in our previous study [59]. Phenylpropanoids, such as lignin and lignans, coumarins, and flavonoids, can function as preformed and inducible antimicrobial compounds or as signal molecules in plant-microbe interactions [60]. The phytohormones, including SA, JA, ET, and ABA, have been well known as important signaling molecular to induce plant defense reactions after pathogens attack [61]. Based on these illustrations, it is surmised that the pathways related to starch and sucrose metabolism, glutathione metabolism, metabolism of xenobiotics by cytochrome P450, biosynthesis of phenylpropanoids, and plant hormones may be significantly modulated in TG9, constituting an important defence against the pathogen attack.

## 5. Conclusion

Global transcriptional profiling was compared between WT and TG9 by hybridizing with Affymetrix Citrus GeneChip in this study. In total, 666 DEGs were identified, including 448 upregulated genes and 218 downregulated genes. After functional annotation and classification, the DEGs implicated in stimulus response and immune system process, cell wall and transcriptional regulation, and cellular and metabolism processes, such as starch and sucrose metabolism, glutathione metabolism, biosynthesis of phenylpropanoids, and plant hormones were hypothesized to play major roles in the canker resistance of TG9. Our data suggest that genetic engineering of a polyamine biosynthetic gene has a profound impact on the transcriptome of the transgenic plants. In the future, extra work is required to verify the function of the DEGs in the canker tolerance of the transgenic line. The present work lays groundwork for deciphering the molecular events of the transgenic line and for tapping desirable genes that hold great potential for genetic engineering aiming at improving biotic stress tolerance. 

## Supplementary Material

Supplemental Table S1: List of significantly upregulated genes in TG9 as comparing with WT. Fold-change = TG9/WT.
Supplemental Table S2: List of significantly downregulated genes in TG9 as compared with WT. Fold-change = TG9/WT.
Supplemental Table S3: KEGG pathways of the upregulated DEGs in TG9.

## Figures and Tables

**Table 1:** Primers pairs used for Genechip verification and expression analysis of candidate genes.

Probe ID	Primers (5′–3′)	Fragment size
Cit.60.1.S1_at	F: TGGCGTATTGGGTGGGGCTG	258 bp
	R: AGATACCCCCGCCCGTGCAA	
Cit.6308.1.S1_at	F: AGCCAGGGCTCGCTTTTGGG	294 bp
	R: TCTTGCATCCAAGCTGACACCAGT	
Cit.19313.1.S1_at	F: GTGAGGTATTTCGGCGAGGGG	348 bp
	R: GGCTTGCGATAACAGAGTGC	
Cit.5367.1.S1_at	F: GACAGCCACATTCCAAGCAG	430 bp
	R: TGAGGCAAGTAGCGACAACG	
Cit.1406.1.S1_s_at	F: TGTTAGGTCTTTTGGTGTCTATTGTT	369 bp
	R: CAGCCTCAGTTTGGGCATTG	
Cit.9510.1.S1_s_at	F: GCTTATGCTTCTCCCAAACGA	450 bp
	R: ACCAGCCAAATACTTGCTCTTC	
Cit.3246.1.S1_at	F: GTGAAAGGAAACGCAACGAA	328 bp
	R: TCCCAGGTCCAGTTACCAATG	
Cit.6097.1.S1_s_at	F: AGCCTATGTCAAAGCAATCCG	391 bp
	R: GCTGCTGTCTACCCCGTCTAA	
Cit.5734.1.S1_at	F: GCTGCTCGCTTTGGCTTCA	367 bp
	R: TTTCTTCATACTCCAGGCACTCA	
Cit.57.1.S1_at	F: GCTGGCGGCGGTGGAGTTAG	408 bp
	R: CGAAAAGCGGCCAACGCTGC	
Cit.8163.1.S1_x_at	F: AGCGTGGAGAAGGCCTGGAC	217 bp
	R: CCGCGAACTGCCCGATCTCC	
Cit.11548.1.S1_at	F: GCCTTACCTTCTCCTTCCTCAT	590 bp
	R: AGTCGGTGGGCAAGTCTCA	
Cit.4425.1.S1_at	F: ACTCCAACACCTTTATTCCTTCAC	337 bp
	R: CATCTCCGCTATTGCCCACT	
Cit.28117.1.S1_s_at	F: TAGACCGACTGACTGCACCAA	239 bp
	R: TGCGAAATACAAAATGAACCC	
Cit.13055.1.S1_at	F: TGACTCCGCCGTTGTGAAGA	253 bp
	R: CACCCGCCGACAACATACA	
Cit.20495.1.S1_at	F: TCACGGACAACGAAGACAAAG	179 bp
	R: TCAACCAAAGCCGAGCAA	
Cit.5856.1.S1_at	F: GAAGCAACAGTTCCAGCAGC	264 bp
	R: CACGAAGCCATCCAGTCAATA	
Cit.17124.1.S1_at	F: CAGAAGGCAGCCACGATGA	299 bp
	R: GATGAGGATGACGAAGAAGAAGC	
Cit.2333.1.S1_at	F: GTGGAAGGGGTAACTGGGATT	461 bp
	R: GCAGAAGTTATTGAAAATGGGTG	
Cit.6121.1.S1_at	F: AGATGAGTCACAAAGACCAGGAGG	205 bp
	R: CACAGGCGTCAACCAATCAAG	
Cit.31932.1.S1_at	F: TGTAGTCGGTGGTGGCTGTAG	436 bp
	R: TGAAAAGTGGGGTGGCATT	
Actin	F: CATCCCTCAGCACCTTCC	190 bp
	R: CCAACCTTAGCACTTCTCC	

**Table 2:** Significantly upregulated genes in response to stimulus and involved in immune system process.

Gene ID	Seq. description	Hit ACC	Fold change
Cit.8878.1.S1_at	Major allergen Pru	ABK06393	8.63139
Cit.11548.1.S1_at	Thaumatin-like protein	Q9SMH2	5.84254
Cit.4426.1.S1_at	Homogentisate geranyl geranyl transferase	XP_002282953	5.63043
Cit.9703.1.S1_at	Beta-1,3-glucanase	CAA03908	5.45386
Cit.29880.1.S1_at	ATP binding	XP_002517441	5.3541
Cit.4504.1.S1_at	Thiamin biosynthesis protein	XP_002525602	4.83609
Cit.14918.1.S1_at	Basic 7s globulin 2 precursor small	XP_002517165	4.6577
Cit.21616.1.S1_at	abc transporter	CBI40242	4.6402
Cit.9706.1.S1_s_at	Beta-1,3-glucanase	ABQ45848	4.54013
Cit.38637.1.S1_at	Protein	XP_002517054	4.25433
Cit.28117.1.S1_s_at	Aspartyl protease family protein	ABK28718	3.93758
Cit.3949.1.S1_s_at	Copper zinc superoxide dismutase	ACC93637	3.85253
Cit.17438.1.S1_at	Protein	XP_002284819	3.69927
Cit.12005.1.S1_s_at	ATP-binding cassette	CBI40242	3.65824
Cit.6364.1.S1_s_at	Peptidase m	XP_002518664	3.64541
Cit.4504.1.S1_s_at	Thiamin biosynthesis protein	XP_002525602	3.60992
Cit.11209.1.S1_s_at	Nematode-resistance protein	XP_002268520	3.47979
Cit.17124.1.S1_at	AP2/ERF domain-containing transcription factor	NP_182011	3.3661
Cit.35636.1.S1_s_at	Hypothetical protein	XP_002262662	3.30867
Cit.9584.1.S1_x_at	Glutathione s-transferase	XP_002273830	3.00065
Cit.9587.1.S1_at	Glutathione s-transferase	XP_002273830	2.91727
Cit.916.1.S1_at	Protein	XP_002525204	2.89418
Cit.31147.1.S1_at	Disease resistance protein	CAN77656	2.82347
Cit.9704.1.S1_at	Beta-1,3-glucanase	ABQ45848	2.79617
Cit.20853.1.S1_at	Citrate synthase	ACU42176	2.79073
Cit.9510.1.S1_s_at	Glutathione s-transferase	XP_002530205	2.75554
Cit.17124.1.S1_s_at	AP2/ERF domain-containing transcription factor	NP_182011	2.72797
Cit.21717.1.S1_at	Wound-induced protein win2	XP_002319077	2.68663
Cit.28472.1.S1_at	Protein	XP_002320004	2.68478
Cit.17374.1.S1_at	Calcium binding protein	ABK06394	2.6752
Cit.23704.1.S1_at	Aspartyl protease family protein	ABK28718	2.64168
Cit.9587.1.S1_x_at	Glutathione s-transferase	XP_002273830	2.62727
Cit.2809.1.S1_s_at	AP2 domain-containing transcription factor	XP_002281709	2.62632
Cit.6280.1.S1_at	bt4 protein binding transcription regulator	XP_002304319	2.60135
Cit.12004.1.S1_at	ATP-binding cassette	CBI30263	2.57256
Cit.12589.1.S1_at	Syntaxin	XP_002326741	2.52495
Cit.32844.1.S1_s_at	Glutathione s-transferase	XP_002520166	2.52047
Cit.31254.1.S1_at	ATP-binding cassette	CAN77838	2.47584
Cit.12560.1.S1_s_at	erd15 protein	XP_002268033	2.45013
Cit.38633.1.S1_at	Transparent testa 12	XP_002314825	2.43996
Cit.24178.1.S1_at	sec12-like protein 1	CBI40184	2.43778
Cit.3550.1.S1_at	Ankyrin repeat-containing	XP_002526791	2.38623
Cit.28117.1.S1_at	Aspartyl protease family protein	ABK28718	2.32863
Cit.9584.1.S1_s_at	Glutathione s-transferase	XP_002273830	2.32824
Cit.13439.1.S1_at	DNA binding	XP_002512121	2.32431
Cit.31932.1.S1_at	Rubisco subunit binding-protein beta	XP_002514548	2.26026
Cit.4131.1.S1_at	Pyridoxal kinase	CBI33550	2.25534
Cit.7994.1.S1_at	Protein	XP_002511077	2.22751
Cit.5117.1.S1_at	Universal stress protein	XP_002515296	2.2129
Cit.4146.1.S1_at	Serine palmitoyltransferase	CBI15735	2.21272
Cit.10854.1.S1_s_at	Protein	XP_002514963	2.20543
Cit.21798.1.S1_at	Glucosyl transferase	ACS87992	2.17486
Cit.465.1.S1_s_at	Thiamin biosynthetic enzyme	XP_002305603	2.17393
Cit.1610.1.S1_at	Peptidyl-prolyl cis-trans isomerase-like protein	XP_002271056	2.17052
Cit.11040.1.S1_at	Hydroxyacylglutathione hydrolase	XP_002329233	2.1672
Cit.2333.1.S1_at	NADPH oxidase	XP_002511059	2.15579
Cit.13055.1.S1_at	Pyruvate kinase	NP_001065749	2.14823
Cit.26113.1.S1_at	Phototropic-responsive nph3 family protein	CAN63893	2.11134
Cit.30576.1.S1_at	Guanylyl cyclase	XP_002277052	2.11123
Cit.836.1.S1_s_at	Protein	XP_002520818	2.09287
Cit.14371.1.S1_at	Homogentisic acid geranylgeranyl transferase	BAH10642	2.08956
Cit.23824.1.S1_at	Protein	XP_002263043	2.08815
Cit.32832.1.S1_at	Protein	XP_002269885	2.07239
Cit.5555.1.S1_at	cop9 complex subunit	XP_002302493	2.05901
Cit.9144.1.S1_at	ATP-dependent clp	XP_002511102	2.05338
Cit.1554.1.S1_at	Membrane protein	CBI27668	2.03856
Cit.35569.1.S1_s_at	Syntaxin	XP_002326741	2.02263
Cit.2495.1.S1_at	RNA binding protein rp120	XP_002511064	2.01664

**Table 3:** Significantly upregulated cell wall-related genes.

Gene ID	Seq. description	Hit ACC	Fold change
Cit.8163.1.S1_x_at	Cysteine proteinase inhibitor	P37842	268.1240
Cit.30421.1.S1_s_at	Cysteine proteinase inhibitor	AAG38521	94.58230
Cit.28011.1.S1_x_at	Cysteine proteinase inhibitor	AAG38521	85.76650
Cit.14918.1.S1_at	Basic 7s globulin 2 precursor small subunit	XP_002517165	4.65770
Cit.35636.1.S1_s_at	Hypothetical protein	XP_002262662	3.30867
Cit.9510.1.S1_s_at	Glutathione s-transferase	XP_002530205	2.75554
Cit.14913.1.S1_at	Class III chitinase	XP_002276365	2.68317
Cit.30421.1.S1_x_at	Cysteine proteinase inhibitor	AAG38521	2.47166
Cit.9064.1.S1_x_at	40s ribosomal protein s9	XP_002511557	2.06885
Cit.9144.1.S1_at	ATP-dependent clp	XP_002511102	2.05338
Cit.2495.1.S1_at	RNA binding protein rp120	XP_002511064	2.01664
Cit.9064.1.S1_s_at	40s ribosomal protein s9	XP_002511557	2.00929

**Table 4:** Significantly upregulated transcription factor related genes.

Gene ID	Seq. description	Hit ACC	Fold change
Cit.17124.1.S1_at	AP2/ERF domain-containing transcription factor	NP_182011	3.36610
Cit.15253.1.S1_s_at	MADS-domain transcription factor	XP_002273223	3.21612
Cit.17124.1.S1_s_at	AP2/ERF domain-containing transcription factor	NP_182011	2.72797
Cit.2809.1.S1_s_at	AP2 domain-containing transcription factor	XP_002281709	2.62632
Cit.6280.1.S1_at	BT4 (BTB and TAZ domain protein 4) protein binding transcription regulator	XP_002304319	2.60135
Cit.39092.1.S1_at	NAC domain protein; IPR003441	XP_002300866	2.30719
Cit.29898.1.S1_at	Unnamed protein product	CBI21863	2.20763
Cit.3005.1.S1_at	FLC-like 1 splice variant 4	ACB72865	2.14257
Cit.8950.1.S1_at	AP2/ERF domain-containing transcription factor	ABB89755	2.13449
Cit.30576.1.S1_at	Guanylyl cyclase	XP_002277052	2.11123
Cit.14044.1.S1_at	Transcription factor, putative	XP_002514876	2.06065
Cit.15941.1.S1_at	Chromatin remodeling complex subunit	ABA18099	2.05183
Cit.35206.1.S1_at	MYB family transcription factor	FR828559	2.00112
